# Patient Information Needs and Decision-Making Before a Cardiac Implantable Electronic Device: A Qualitative Study Utilizing Social Media Data

**DOI:** 10.1007/s10880-024-10024-6

**Published:** 2024-05-21

**Authors:** Mitchell Nicmanis, Joshua Holmes, Melissa Oxlad, Anna Chur-Hansen

**Affiliations:** 1 School of Psychology, Faculty of Health and Medical Sciences, The University of Adelaide, Level 5, Hughes Building North Terrace Campus, Adelaide, SA 5000 Australia; 2 School of Computer and Mathematical Sciences, Faculty of Sciences, Engineering and Technology, The University of Adelaide, Adelaide, SA Australia

**Keywords:** Cardiac implantable electronic device, Decision-making, Qualitative, Implantable cardioverter-defibrillator, Pacemaker

## Abstract

The decision to receive a cardiac implantable electronic device (CIED) represents a challenging experience for patients. However, the majority of previous research has only considered retrospective accounts of patient experiences. This study aimed to use social media data to characterize the information sought by people anticipating or considering CIED implantation and factors that influence their decision-making experiences. A Python-based script was used to collect posts made to a community intended for discussions concerning CIEDs on the social media platform Reddit. Reflexive content analysis was used to analyze the collected data. From 799 posts collected, 101 made by 86 participants were analyzed. The reported median (range) age of participants was 34 (16–67), and most were anticipating or considering a pacemaker. Three overarching categories classified the data: “Use of social media to meet informational and other needs”; “Factors influencing acceptance of the need for implantation”; and “Specific concerns considered during decision-making.” Participants anticipating or considering a CIED predominantly sought experiential information. Among asymptomatic participants, doubts were prevalent, with acceptance being an influential factor in decision-making. Healthcare professionals should recognize the informational and emotional needs of prospective CIED patients and tailor support mechanisms to better facilitate their decision-making.

**Supplementary Information:**

The online version contains supplementary material available at 10.1007/s10880-024-10024-6.

## Introduction

When faced with potentially life-altering treatment choices, such as those necessitated by heart failure, the decision to receive a cardiovascular intervention is both difficult and stressful (Hamel et al., 2018). Cardiac implantable electronic devices (CIEDs) are an effective intervention for a range of cardiac conditions, including arrhythmia, heart failure, and cardiac arrest (Al-Khatib et al., 2018; Atherton et al., 2018; Glikson et al., 2021; Kusumoto et al., 2019; Zeppenfeld et al., 2022). Deciding whether to be implanted with one of these devices is challenging, requiring people to balance possible treatment burdens, complication risks, and their own mortality (Carroll et al., 2013, 2018; Green et al., 2016; Lewis et al., 2014; Matlock et al., 2011; Ottenberg et al., 2014). Some people who have undergone implantation may even come to regret their initial decision (Varghese et al., 2020).

Limited research has considered the decision-making experiences associated with CIED implantation (Malecki-Ketchell et al., 2017; Ottenberg et al., 2014). The majority of research has focused on decisions regarding implantable cardioverter-defibrillators (Barakat et al., 2020; Carroll et al., 2013, 2018; Chan et al., 2016; Green et al., 2016; Lewis et al., 2014; Matlock et al., 2011), with other device types receiving less attention (Ottenberg et al., 2014; Thomas, 2012). Additionally, current research has largely relied on patients’ retrospective accounts of implantation decision-making (Barakat et al., 2020; Carroll et al., 2013; Chan et al., 2016; Green et al., 2016; Lewis et al., 2014; Matlock et al., 2011; Ottenberg et al., 2014; Varghese et al., 2020), with few studies including prospective participants or those who have deferred their decision (Carroll et al., 2018; Thomas, 2012). Based on current research, there is a need to gain insight into the experiences of people anticipating or considering CIED implantation to better understand and aid patient decision-making.

The underrepresentation of prospective patients anticipating or considering CIED implantation in research may stem from challenges in accessing this population. These challenges encompass ethical concerns about influencing patient choices, coupled with the fact that these devices are frequently implanted under urgent or emergency conditions (Dell’Era et al., 2022; Ranasinghe et al., 2019). Publicly available social media data present a promising solution to this issue, as it provides researchers the opportunity to investigate the candidly expressed perspectives of populations that are challenging to engage through traditional methods (Smedley & Coulson, 2021). Additionally, social media has had an increasing role in patient decision-making (Patrick et al., 2022; Smailhodzic et al., 2016) and information from these sources may play a role in decreasing the anxiety of implantable cardioverter-defibrillator patients (Richards et al., 2016). Therefore, this study aimed to use qualitative data collected from social media to characterize the information sought by people anticipating or considering CIED implantation and the factors that influence their decision-making experiences.

## Methods

### Design and Data Source

This study utilized a descriptive qualitative methodological approach to analyze a data corpus of posts made to a community hosted on the social media platform Reddit. Users of this platform with an account post content to user-moderated thematically curated communities called “subreddits.” Data were collected from a subreddit intended for the discussion of CIEDs identified by a previous study (Nicmanis et al., 2023). Participants were people who indicated they were anticipating or considering CIED implantation as a treatment option. At the time of data collection (April 16, 2023), the subreddit consisted of 1600 members, 799 posts, and 7496 comments.

Reddit was specifically chosen as the data source for this study for several reasons. Firstly, the thematically curated nature of subreddits has been used extensively to explore specific topics of interest (Proferes et al., 2021). These subreddits have become desirable places for people to share health-related information, particularly concerning the management of chronic diseases and heart conditions (Foufi et al., 2019; Nicmanis et al., 2023; Proferes et al., 2021). Secondly, Reddit has an existing programming infrastructure that allows for the accurate collection of data. Finally, Reddit’s pseudo-anonymous nature and established data collection conventions minimize the ethical concerns typically associated with research using other social media sites or private forums.

### Ethical Considerations and Approval

This study received ethical approval from the University of Adelaide School of Psychology Human Research Ethics Sub-Committee (approval number: 22/27). Although there is no widely agreed-upon standard for ethical practices in researching publicly available online data (Golder et al., 2017), it is imperative to avoid causing harm to social media communities and users (Smedley & Coulson, 2021). Consequently, the subreddit used for this study was publicly accessible and did not have any rules prohibiting data collection. Furthermore, we removed all personally identifiable information from reported extracts, and assigned each participant a numeric pseudonym.

### Data Collection and Screening

In order to collect posts from the subreddit in a manner consistent with Reddit’s Terms of Service (Proferes et al., 2021), a script utilizing the Python Reddit Application Programming Interface Wrapper (PRAW; Boe, 2022) was used. The use of PRAW allowed for the systematic and sequential collection of each post made to the subreddit since its inception. The second author collected the data, and the first author screened each post. Only posts made by participants who, at the time of posting, clearly stated that they were anticipating or considering CIED implantation were included. Where data could be ascertained from the included posts, a register of participant demographic information was created that included the declared age, gender, anticipated CIED type, and cardiac conditions of participants. Each username was assumed to represent a unique participant.

### Data Analysis

A reflexive adaptation of conventional qualitative content analysis (Nicmanis, 2024), as described by Hsieh and Shannon (2005), was used to inductively classify data from the collected posts into a hierarchical structure of codes, subcategories, and categories. The following research question guided the analysis: “What information is sought by people anticipating or considering CIED implantation, and what are potential factors that influence their decision-making experiences?” For each category, subcategory, and code, frequencies of occurrence were calculated by dividing the number of participants who had produced corresponding textual data by the total number of participants. These frequencies were used to bolster the credibility of the analysis. It was believed that the sample of posts was sufficient to contribute to current literature (Malterud et al., 2016) as previous studies have predominantly considered retrospective accounts of decision-making (Barakat et al., 2020; Carroll et al., 2013; Chan et al., 2016; Green et al., 2016; Lewis et al., 2014; Matlock et al., 2011; Ottenberg et al., 2014; Varghese et al., 2020). The first author, a male PhD student, conducted the analysis using the NVivo 12 Plus software. The third and fourth authors (experienced health psychologists with prior research and clinical experience working with cardiac patients) reviewed the analysis.

## Results

### Post Characteristics and Participant Demographic Information

The Python script collected 799 posts from the subreddit; of these, 698 were removed as they were not made by people anticipating or considering CIED implantation. The final sample consisted of 101 posts made by 86 participants. The posting dates of included posts ranged from July 2018 to April 2023, with greater than 90% of the posts being from January 2020 onwards. The 58 participants who reported their age were aged between 16 and 67 (median 34) years. Table 1 illustrates the reported demographic characteristics of participants.

**Table 1:** Participant demographic information

Characteristic	Value	%
Gender		
Male	18	20.9
Female	19	22.1
Not reported	49	57.0
Anticipated device type		
Permanent pacemaker	53	61.6
Implantable cardioverter-defibrillator	19	22.1
Subcutaneous implantable cardioverter-defibrillator	6	7.0
Cardiac-resynchronization therapy device	3	3.5
Specific CIED yet to be determined	2	2.3
Leadless Pacemaker	1	1.2
Not determinable	2	2.3
Reported need for a device		
Currently scheduled for implantation	40	46.5
Advised they need a CIED	21	24.4
Advised that they may eventually need a CIED	15	17.4
Has not had a CIED officially mentioned to them	6	7.0
Seeking a second opinion after being declined	2	2.3
Unclear	2	2.3

*CIED* cardiac implantable electronic device

Table 2 illustrates the reported diagnosed or undiagnosed cardiac conditions that participants attributed to their need to anticipate or consider a CIED.

**Table 2:** Cardiac conditions reported by participants

Characteristic	Value	%
Cardiac condition		
AV block	17	19.8
Various bradyarrhythmias	13	15.1
Various cardiomyopathies	11	12.8
Atrial fibrillation	7	8.1
Unspecified arrhythmia	6	7.0
Heart failure	5	5.8
Various tachyarrhythmias	5	5.8
Left bundle branch block	4	4.7
Congenital conditions	3	3.5
Long QT syndrome	3	3.5
Tachycardia-bradycardia syndrome	3	3.5
Autonomic dysfunction	2	2.3
Brugada syndrome	2	2.3
Right bundle branch block	2	2.3
Syncope	2	2.3
Atrial flutter	1	1.2
Cardioinhibitory reflex syncope	1	1.2
Postural tachycardia syndrome	1	1.2
Sick sinus syndrome	1	1.2
Sinus pause	1	1.2
Sudden cardiac arrest	1	1.2
Further details		
Participants with cardiac multimorbidity	15	17.4
Condition not stated	13	15.1

### Analysis Structure

**Fig. 1:**
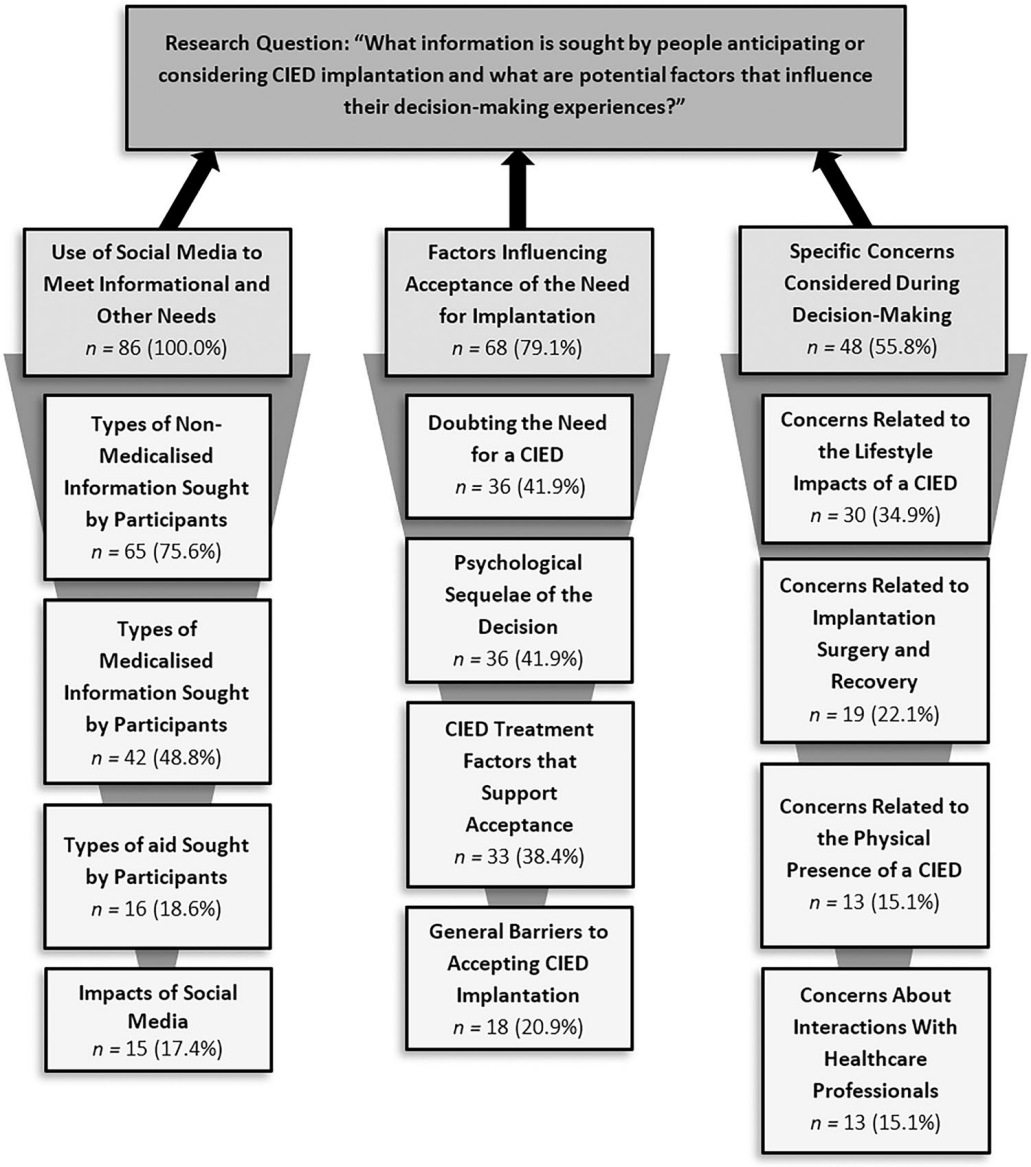
Analysis structure including categories (gray) and subcategories (light gray). *CIED* cardiac implantable electronic device; *n* the occurrence frequency calculated based on the number of participants anticipating or considering a CIED who had produced corresponding textual data for each strata of the analysis

The posts were classified into 77 codes, 12 subcategories, and 3 overarching categories that described the information sought by people anticipating or considering a CIED and the potential factors influencing their decision-making experiences (Fig. 1). See the Supplementary Materials, Table S1, for the full analysis structure. Table 3 provides example quotes from participants for each subcategory and frequencies of occurrence calculated for each subcategory and category based on the number of participants who had produced corresponding textual data.

**Table 3:** Example quotes from participants for each subcategory with occurrence frequencies

Categories and subcategories	Example quote	*n* (%)
Use of Social Media to Meet Informational and Other Needs		86 (100)
Types of Non-Medicalized Information Sought by Participants	“My doctor advised an ICD [implantable cardioverter-defibrillator]. And I am completely torn on what to do … I’m wondering if we have any members in similar situations and how they proceeded.” (Participant 51) “Looking for any advice or experience with recovery and generally in life that may help?” (Participant 67)	65 (75.6)
Types of Medicalized Information Sought by Participants	“Should I pursue a pacemaker with that low of a heart rate [48 bpm] lying down?” (Participant 8) “I was wondering how slow your resting heart rate could be to be offered a pacemaker. My resting heart rate is 56 bpm and 49 while sleeping.” (Participant 32)	42 (48.8)
Types of Aid Sought by Participants	“I have no one to talk to so I figured I’d randomly blurt stream-of-consciousness thought out about how I’m feeling right now to random Internet strangers.” (Participant 38) “I just need someone to reassure me that getting a pacemaker at 22 is no big deal.” (Participant 81)	16 (18.6)
Impacts of Social Media	“It really helped [subreddit responses regarding whether to get a device] my wife and I decide which way to go. … Anyways, so now it’s [CIED implantation] scheduled.” (Participant 9) “I’m a bit nervous but looking through this sub [subreddit], it seems like most people are able to get back to normalcy pretty soon!” (Participant 82)	15 (17.4)
Factors Influencing Acceptance of the Need for Implantation		68 (79.1)
Doubting the Need for a CIED	“I’m a healthy 26-year-old male, no family history of heart problems, and a top athlete. Should I get a second opinion? I am very hesitant to get a pacemaker put in.” (Participant 37) “I’m having a hard time justifying why I would want this thing when it won’t improve my condition and ‘might’ save my life, but then also might not.” (Participant 41)	36 (41.9)
Psychological Sequelae of the Decision	“I am scared. I feel like I’m just giving up by getting it done. Maybe I am in denial and hopeful someone will call and say hey we figured it out you are going to be fine.” (Participant 58) “I’m terrified of the thought of an inappropriate shock or it impacting my life and mental health in a really negative way.” (Participant 78)	36 (41.9)
CIED Treatment Factors that Support Acceptance	“Unlike most people I wasn’t taken aback when I got the diagnosis. To me it’s just another step on my way to becoming the 6-million-dollar man.” (Participant 42)	33 (38.4)
General Barriers to Accepting CIED Implantation	“I know the pacemaker will help and hopefully increase my quality of life but making such a permanent decision is very scary for me.” (Participant 68)	18 (20.9)
Specific Concerns Considered During Decision-Making		48 (55.8)
Concerns Related to the Lifestyle Impacts of a CIED	“I really do not want this surgery to change my life in any way because there’s nothing negative in it now … I just don’t want to go from feeling fine and wonderful to sick, you know?” (Participant 38)	30 (34.9)
Concerns Related to Implantation Surgery and Recovery	“I’m of course nervous about it and focusing on what I need to do to be prepared. [lists complex commitments] … I do wish I could stay overnight and have everything taken care of for me without guilt.” (Participant 4)	19 (22.1)
Concerns Related to the Physical Presence of a CIED	“Will it protrude from wherever it’s placed, like how visible is an ICD [implantable cardioverter-defibrillator] when it’s in your body? … Is it uncomfortable, or is it just something you get used to over time?” (Participant 30)	13 (15.1)
Concerns about Interactions with Healthcare Professionals	“It seems strange and overkill to me that this should be the first thing the doctor would go to in my situation… Seems fishy to me.” (Participant 16) “Anyone else feel like their doc didn’t really care because of their age and sex? Please tell me I am not crazy for going to another doc?” (Participant 50)	13 (15.1)

Occurrence frequencies were calculated by dividing the number of participants who had produced corresponding textual data (*n*) by the total number of participants (*N* = 86) for each category and subcategory

*CIED* cardiac implantable electronic device

#### Use of Social Media to Meet Informational and Other Needs

The first category provided insights into participants’ use of the subreddit to meet their informational and other needs. Within this category, the first subcategory captured the non-medicalized and experiential information participants sought by using the subreddit. Participants wanted to learn about others’ implantation experiences, receive advice, answer questions about living with a CIED, see photographs of other users’ insertion sites, and hear about others’ success stories. Participants looked for people in similar situations and drew on others’ experiences to determine their course of action.

The second subcategory captured the medicalized information sought by participants. Information sought included technical questions about implantation surgery and recovery, leadless pacemakers, the impacts of a CIED on their clinical condition, the time between diagnosis and implantation, and the implantation capabilities of specific hospitals. In addition, nine participants asked other users whether their symptoms necessitated the implantation of a CIED.

The third subcategory described the ways in which participants used the subreddit as a source of tangible aid. Participants visited the subreddit seeking reassurance, and the ability to “vent” to other users emerged as a valuable form of support, especially for those without other avenues of assistance.

The fourth subcategory summarized how the subreddit impacted participants and their experiences. While three participants expressed that posts on the subreddit had made them anxious, 13 reported that reading about others’ experiences was beneficial. In the latter case, this could be a determining factor in accepting implantation.

#### Factors Influencing Acceptance of the Need for Implantation

The second category reflected subcategories related to factors influencing participants’ acceptance of the need for a CIED. The first subcategory captured instances where participants indicated that they doubted the need to be implanted with a CIED. This doubt was largely attributed to currently feeling healthy, not being adversely impacted by their symptoms, viewing themselves as too young, and having no family history of cardiac illnesses. Alongside these mismatches between medical needs and participants’ perceived health status, 13 participants made general expressions of doubt, and 2 participants expressed difficulty accepting a device as it may not improve their clinical condition.

The psychological sequelae of the decision acted as a potential barrier to accepting implantation, descriptions of which were summarized in the second subcategory. Being told that they must undergo implantation was confronting, with participants reporting feelings of being lost or confused, and some experienced a sense of denial. Participants indicated for implantable cardioverter-defibrillator implantation expressed anxiety about potential shocks. Prominently, participants expressed fear and anxiety that contributed to the difficulty of accepting implantation.

The third subcategory focused on CIED treatment factors that supported acceptance. Participants considered adverse symptom burdens undesirable and expressed a desire for a CIED to enhance their quality of life, viewing these devices as a source of protection. Additionally, participants desired a CIED because this appeared to be the best alternative compared to other treatments and because they perceived benefits from specific device types. For participants experiencing extensive symptom burdens, previous surgical experiences were reported to decrease fears, and four participants viewed a CIED as an inevitability of their treatment.

The fourth subcategory contained posts concerning general barriers to accepting CIED implantation. Participants expressed concerns about the implantation surgery due to a variety of reasons: they had no prior experience with surgeries, felt underinformed about the procedure, had experienced medical trauma, did not know others with cardiac conditions, and were apprehensive that the device would remind them of their cardiac condition. These devices were perceived as challenging to adapt to, and the decision to be implanted with a CIED was most often seen as a serious commitment.

#### Specific Concerns Considered During Decision-making

The last category captured participants’ specific concerns about implantation surgery and living with a CIED that they considered during their decision-making. The most frequent subcategory contained concerns related to the lifestyle impacts of a CIED. Concerns included the impacts of a CIED on participants’ physical activity, potential risks from electromagnetic interference, and implications for employment, parenting abilities, driving capabilities, finances, medication requirements, and life expectancy. Additionally, participants expressed concerns about the impacts of battery replacements. Four participants were concerned that the impacts of a CIED may cause declines in their quality of life.

The second subcategory summarized a diverse range of concerns related to implantation surgery and recovery. Participants expressed concerns about who performs the implantation surgery, sleeping while recovering, pain, infections, diet, and maintaining contact with their healthcare professionals. Participants with complex health needs and commitments expressed concerns regarding the logistics of procedures. In these cases, specific concerns included managing recovery times, problems with their current living situations, uncertainty regarding overnight hospital stays, being alone, burdening friends, maintaining contact with clinicians, and emergency contacts.

The third subcategory described participants’ concerns related to the physical presence of the CIED once implanted. These included concerns about the device’s size, being able to feel the CIED inside them, the device protruding, rubbing from external objects, and body image changes. Participants also expressed concerns about physical failures of device leads. They were most concerned about comfort and the device being as discreet as possible.

The final subcategory captured participants’ concerns about their interactions with healthcare professionals. Participants were concerned about conflicting decisions between their different healthcare professionals and other breakdowns in communication that led to confusion. Participants most frequently raised concerns about their healthcare professionals’ judgments and expressed doubt or even suspicion regarding their treatment decisions.

## Discussion

We aimed to characterize the information sought by people anticipating or considering implantation with a CIED and factors that potentially influenced their decision-making. Participants predominantly sought experiential information about implantation surgery and what to expect after being implanted with a device. Beyond informational needs, participants drew on the subreddit analyzed as a resource to receive reassurance and express their difficulties. Contemplating the decision to have a CIED implanted was deeply emotional and complex, with the desire of participants for a device heavily influenced by doubts and acceptance. Additionally, participants deliberated on various concerns related to the procedure and the experience of living with a device. These findings primarily reflect the perspectives of younger individuals anticipating or considering pacemaker implantation. While these results share similarities with previous research that has employed retrospective surveys and interviews to examine decision-making (Barakat et al., 2020; Carroll et al., 2013; Chan et al., 2016; Green et al., 2016; Lewis et al., 2014; Matlock et al., 2011; Ottenberg et al., 2014; Varghese et al., 2020), the use of social media data has provided new insights.

The role of doubt and acceptance in participants’ willingness to undergo implantation was particularly noteworthy. Those who were young, asymptomatic, and physically active expressed doubts over their need for a device. Doubts could be so great that some participants expressed suspicion toward their healthcare professionals’ practice. Conversely, those who experienced adverse symptom burdens were more accepting of device implantation and viewed it as a way to improve their health. Concerns about health status have featured as influential factors for decision-making in previous literature. People have generally expressed a desire to avoid death (Carroll et al., 2018; Lewis et al., 2014; Matlock et al., 2011), and some balanced this against the possible impacts of a device on their quality of life (Carroll et al., 2018; Matlock et al., 2011). Poorer pre-implantation health has been seen to increase the immediacy of decisions (Carroll et al., 2013), and people who are asymptomatic have reported seeing a device as unnecessary (Matlock et al., 2011; Ottenberg et al., 2014). Our study extends previous literature by highlighting that the interplay between acceptance and doubt, especially concerning medical necessity, influences the decision-making of individuals anticipating or considering device implantation.

Previous studies have generally considered device-related acceptance in post-implantation contexts through measures such as the Florida Patient Acceptance Survey (Burns et al., 2005). This measure provides possibly the only theoretical operationalization of CIED-related acceptance, and high acceptance levels have demonstrated associations with better mental and quality of life outcomes (Bedair et al., 2015; Burns et al., 2005). However, a comprehensive theoretical understanding of CIED-related acceptance spanning from the pre-implantation to post-implantation period is absent from the current literature. Greater understanding of patient acceptance and doubt could enhance patient education and better support people who are asymptomatic in their decision-making.

The use of social media data has allowed insights into patient decision-making processes and concerns possibly overlooked by current research. Existing evidence regarding CIEDs and heart failure treatment decisions indicates that people take on either “passive” or “active” decision-making styles (Lewis et al., 2014; Malecki-Ketchell et al., 2017; Matlock et al., 2010). Active decision-makers proactively seek clinical information, solicit opinions, and weigh the pros and cons of a treatment. In contrast, passive decision-makers rely on healthcare professionals (Green et al., 2016; Matlock et al., 2011) or family members (Malecki-Ketchell et al., 2017) to make choices on their behalf. Most posts made to the subreddit were information-seeking, suggesting that participants were active decision-makers. Conversely, the pseudo-anonymity granted by social media may have encouraged passive decision-makers to voice their concerns about implantation. Regardless, the anonymity afforded to participants enabled them to communicate more openly than they may with healthcare professionals or researchers. Participants described the immense psychological stresses they faced, the challenges they experienced with healthcare professionals, and a range of often highly specific concerns.

Similarly, using social media data has demonstrated the potential value of peer support for people anticipating or considering a CIED, as most participants posted to the subreddit to gain experiential information. The definition of a “peer” has varied in research (Thompson et al., 2022), but they are generally considered to be people who share situational similarities with another (Shalaby & Agyapong, 2020). Peer support is typically categorized as either formal, structured and mediated as a healthcare intervention, or informal, arising organically within the community or among patients (Fortuna et al., 2019; Shalaby & Agyapong, 2020). Most research focusing on peer support in cardiovascular healthcare has assessed formalized interventions with mixed results (Clayton et al., 2019; Parry & Watt-Watson, 2010).

Our study indicates that informal online peer support may be both desired by people anticipating or considering a CIED and potentially beneficial in aiding their decision-making. However, online forums are not without risk, with a study examining the accuracy of information shared on heart failure-related health forums reporting minimal use of evidence-based information (Farnood et al., 2022). This finding is especially concerning as several participants visited the subreddit to determine the necessity of a device for their condition. Further work is needed to explore the utility of formal and informal peer support for this population, and healthcare professionals must consider ways to minimize the risk of misinformation.

### Implications

Although the participants in this study were not representative of the typical demographic considering CIED implantation, these findings nonetheless highlight potential areas for enhancing the information provided to patients. Participants desired more information about the experiences of receiving and living with these devices. Discussions that consider values and outcomes following a medical intervention are a key element of shared decision-making processes (Chung et al., 2021; Rao et al., 2022). Healthcare professionals should explore ways of providing greater information about experiential and quality of life outcomes during clinical interactions. They should also explore facilitating peer interactions in ways that mitigate the risks of privacy breaches (Fortuna et al., 2019) and misinformation (Farnood et al., 2022) inherent in unmoderated social media. Supporting this, further research should explore the experiences of a representative sample of individuals considering CIED implantation.

In addition, there is a need to better inform people who are asymptomatic about their options for device implantation. Not experiencing symptoms can cast doubt on the utility of a CIED, even when it may be medically necessary. Healthcare professionals have a duty to effectively communicate these potential risks to patients, helping them to gauge whether a treatment aligns with their needs (Freeman, 2019). In clinical discussions with patients who are asymptomatic, healthcare professionals must clearly convey the biomedical rationale for a CIED and actively address any doubts. Additionally, healthcare professionals should explore innovative approaches to better inform asymptomatic patients. Such approaches could include the development of psychoeducational materials tailored for patients who are asymptomatic or facilitating peer interaction or knowledge sharing with others who have faced similar decisions.

### Limitations

The use of social media data provided novel insights into the experiences of individuals anticipating or considering CIED implantation. However, social media data present limitations that must be considered when interpreting our findings. Data collected from social media inherently reflects a bias toward individuals who are inclined to share their experiences openly and possess a higher degree of technological literacy. These factors introduce significant selection bias challenges. Reddit users are more likely to be from English-speaking backgrounds, younger, and male than the general population (Amaya et al., 2021). The participants in this study were also younger, potentially more anxious, and more frequently considering pacemaker implantation, compared to the typical demographic undergoing CIED implantation. Furthermore, the characteristics of Reddit usage suggest that our participants may represent a distinct subpopulation, potentially turning to online platforms for information in response to insufficient guidance from their healthcare professionals. The findings, therefore, primarily represent the views of this subgroup of social media users and not the experiences of general clinical populations. Finally, as participants could not be contacted, it was not possible to validate or more deeply explore the information collected.

## Conclusion

We used social media data to provide insights into the experiences of individuals anticipating or considering the implantation of a CIED, a population previously underrepresented in research. The results of this study primarily reflect the perspectives of younger participants considering pacemaker implantation. For participants, deciding whether to be implanted with one of these devices is complex and emotionally difficult, especially for asymptomatic individuals who were hesitant to accept their need for a device. Participants in our study primarily requested experiential information about the procedure and life post-implantation. Consequently, social media websites, such as Reddit, may represent desirable forums for these discussions due to a potential lack of accessible literature on this topic or the inadequate translation of complex medical terms by healthcare professionals. It is imperative that patients are comprehensively informed and that their doubts are duly addressed. Considering participants’ desire for experiential knowledge, healthcare professionals should educate their patients about the day-to-day realities of living with these devices and consider support interventions.

## Supplementary Information

Below is the link to the electronic supplementary material.

Supplementary file1 (PDF 127 KB)

## Data Availability

Data are available from the corresponding author on reasonable request.
