# Supplementary material for: Patient Information Needs and Decision-Making Before a Cardiac Implantable Electronic Device: A Qualitative Study Utilizing Social Media Data
Source: J Clin Psychol Med Settings. 2024 May 21;32(1):121–30. doi: 10.1007/s10880-024-10024-6 (PMC11914295; doi:10.1007/s10880-024-10024-6)
Supplement: Supplementary file 1 — Supplementary file1 (PDF 127 KB) [file 10880_2024_10024_MOESM1_ESM.pdf]

Supplementary Material

Table S1

Full Analysis Structure With Occurrence Frequencies Calculated Based on the Total Number of Participants (N=86) Anticipating or Considering a Cardiac Implantable Electronic Device (CIED)

Categories	Subcategories	Codes	n	% of N
Use of Social Media to Meet Informational and Other Needs			86	100.0
	Types of non-medicalised information and aid sought by participants		65	75.6
		Seeking the experiences of others	24	27.9
		Seeking advice about living with a CIED	19	22.1
		Seeking answers to questions about CIEDs and living with them	18	20.9
		Seeking people who have been in similar situations	18	20.9
		Seeking experiences with implantation surgery and recovery	15	17.4
		Seeking general tips about living with a CIED	6	7.0
		Seeking experiences with assistive technologies	3	3.5
		Seeking photos of other users' CIED insertion sites	2	2.3
		Seeking the success stories of other patients	2	2.3
	Types of medicalised information sought by participants		42	48.8
		Questions about the specifics of surgery and recovery	23	26.7
		Asking the subreddit if they need a CIED	9	10.5
		Questions about leadless pacemakers	6	7.0
		Questions about the impacts of a CIED on their clinical condition	6	7.0

		Questions about time between diagnosis and implantation	3	3.5
		Questions about the capabilities of specific hospitals	1	1.2
	Types of aid sought by Participants		16	18.6
		Using the subreddit to vent about their experiences	11	12.8
		Seeking reassurance about the current problems they face	6	7.0
	Impacts of social media		15	17.4
		Reading about others' experiences has helped them decide	13	15.1
		Reading about other's experiences has made it harder to decide	3	3.5
Factors influencing acceptance of the need for implantation			68	79.1
	Doubting the need for a CIED		36	41.9
		Difficulty accepting as they currently feel healthy	17	19.8
		Difficulty accepting as they have not been impacted by symptoms	15	17.4
		Questioning whether they need a CIED	13	15.1
		Difficulty accepting as they view themselves as too young	10	11.6
		Difficulty accepting due to no prior family history of cardiac illness	3	3.5
		Difficulty accepting as a CIED may not improve their condition	2	2.3
	Psychological sequelae of the decision		36	41.9
		Feeling scared about having a CIED implanted	20	23.3
		Feeling anxious about having a CIED implanted	14	16.3
		Feeling lost or confused about their current situation	8	9.3
		Feeling anxiety about potential shocks from an ICD	3	3.5
		Feeling as if they are experiencing denial	2	2.3

	CIED treatment factors that support acceptance		33	38.4
		living without an CIED is undesirable due to symptom burden	12	14.0
		Viewing a CIED as providing protection from cardiac events	10	11.6
		Worried that their unmanaged condition will harm or kill them	9	10.5
		Viewing a CIED as a way of improving quality of life	7	8.1
		Desires a specific CIED due to perceived benefits	5	5.8
		Viewing the CIED as an inevitability of their treatment	4	4.7
		Viewing a CIED as the best alternative to other treatments	3	3.5
		Prior surgical experiences decrease worry	2	2.3
	General barriers to accepting CIED implantation		18	20.9
		Viewing implantation as a serious commitment	5	5.8
		Worried about implantation as they have not had previous surgeries	4	4.7
		Feeling that they have not been provided with information	3	3.5
		Has experienced trauma from previous medical experiences	2	2.3
		Worried as they don't know anyone else with a heart condition	2	2.3
		Living with a CIED will remind them of their heart condition	1	1.2
		Hesitant to accept as worried about adapting to the device	1	1.2
Specific concerns considered during decision making			48	55.8
	Concerns related to the lifestyle impacts of a CIED		30	34.9
		Concerned about impacts on their physical activity	10	11.6

		Concerned about the impacts on their lifestyle	8	9.3
		Concerned about electromagnetic interference	5	5.8
		Concerned about impacts on their employment	5	5.8
		Concerned about impacts on parenting	5	5.8
		Concerned about declines in quality of life after implantation	4	4.7
		Concerned about impacts on driving	4	4.7
		Concerned about associated financial costs	3	3.5
		Concerned about battery replacement	2	2.3
		Concerned about medications	1	1.2
		Concerned about reduced life expectancy	1	1.2
	Concerns related to implantation surgery and recovery		19	22.1
		Concerned about recovery times	5	5.8
		Concerned about sleeping while recovering	4	4.7
		Concerned about pain from the surgery	3	3.5
		Concerned about the impacts of recovery on their living situation	3	3.5
		Concerned about the uncertainty of overnight hospital stays	3	3.5
		Concerned about being alone while recovering	2	2.3
		Concerned about infections	2	2.3
		Concerned about burdening their friends during recovery	1	1.2
		Concerned about diet during recovery	1	1.2
		Concerned about emergency contacts during recovery	1	1.2
		Concerned about maintaining contact with clinicians	1	1.2
		Concerned about who will perform the implantation surgery	1	1.2

	Concerns related to the physical presence of a CIED		13	15.1
		Concerned about feeling the presence of the CIED inside them	7	8.1
		Concerned about the device protruding out of their body	5	5.8
		Concerned about body image changes after implantation	4	4.7
		Concerned about device leads and lead failures	4	4.7
		Concerned about external objects rubbing against the CIED	2	2.3
		Concerned about the size of CIEDs	2	2.3
	Concerns about Interactions with healthcare professionals		13	15.1
		Concerned about their clinicians judgements	8	9.3
		Concerned about conflicting decisions between clinicians	5	5.8
		Concerned about communication from their clinicians	4	4.7
